# Amoebae as Potential Environmental Hosts for *Mycobacterium ulcerans* and Other Mycobacteria, but Doubtful Actors in Buruli Ulcer Epidemiology

**DOI:** 10.1371/journal.pntd.0001764

**Published:** 2012-08-07

**Authors:** Sophie Gryseels, Diana Amissah, Lies Durnez, Koen Vandelannoote, Herwig Leirs, Johan De Jonckheere, Manuel T. Silva, Françoise Portaels, Anthony Ablordey, Miriam Eddyani

**Affiliations:** 1 Evolutionary Ecology Group, University of Antwerp, Antwerp, Belgium; 2 Noguchi Memorial Institute of Medical Research, University of Ghana, Legon, Ghana; 3 Institute of Tropical Medicine, Antwerp, Belgium; 4 Danish Pest Infestation Laboratory, Department of Agro-ecology, Aarhus University, Slagelse, Denmark; 5 Research Unit for Tropical Diseases, de Duve Institute, Université Catholique de Louvain, Brussels, Belgium; 6 Scientific Institute of Public Health, Brussels, Belgium; 7 Institute for Molecular and Cell Biology (IBMC), University of Porto, Porto, Portugal; University of Tennessee, United States of America

## Abstract

**Background:**

The reservoir and mode of transmission of *Mycobacterium ulcerans*, the causative agent of Buruli ulcer, remain unknown. Ecological, genetic and epidemiological information nonetheless suggests that *M. ulcerans* may reside in aquatic protozoa.

**Methodology/Principal Findings:**

We experimentally infected *Acanthamoeba polyphaga* with *M. ulcerans* and found that the bacilli were phagocytised, not digested and remained viable for the duration of the experiment. Furthermore, we collected 13 water, 90 biofilm and 45 detritus samples in both Buruli ulcer endemic and non-endemic communities in Ghana, from which we cultivated amoeboid protozoa and mycobacteria. *M. ulcerans* was not isolated, but other mycobacteria were as frequently isolated from intracellular as from extracellular sources, suggesting that they commonly infect amoebae in nature. We screened the samples as well as the amoeba cultures for the *M. ulcerans* markers IS*2404*, IS*2606* and KR-B. IS*2404* was detected in 2% of the environmental samples and in 4% of the amoeba cultures. The IS*2404* positive amoeba cultures included up to 5 different protozoan species, and originated both from Buruli ulcer endemic and non-endemic communities.

**Conclusions/Significance:**

This is the first report of experimental infection of amoebae with *M. ulcerans* and of the detection of the marker IS*2404* in amoeba cultures isolated from the environment. We conclude that amoeba are potential natural hosts for *M. ulcerans*, yet remain sceptical about their implication in the transmission of *M. ulcerans* to humans and their importance in the epidemiology of Buruli ulcer.

## Author Summary

Buruli ulcer (BU) is a devastating skin disease caused by *Mycobacterium ulcerans*, an environmental bacterium that is probably linked to slow-running water. It is unlikely to occur free-living, but even though *M. ulcerans* DNA has been detected in quite a few different organisms (with most studies focusing on insects), it is still not clear what its real reservoir is. Amoeboid protozoa, inhabitants of biofilms in slow flowing water, are good candidates since all previously tested mycobacteria are resistant to the digestion by these macrophage-like organisms. In this paper we demonstrate that *M. ulcerans* can indeed infect Acanthamoeba polyphaga in the lab, and remain viable intracellularly. We also collected water, biofilm and detritus samples in BU endemic and non-endemic regions in Ghana. We found that several mycobacteria species commonly occur intracellularly in protozoa in these environments. Amoebae were isolated from almost all samples, and an *M. ulcerans* marker (IS*2404*) was detected in 4% of the amoeba cultures. We conclude that amoebae are potential hosts for *M. ulcerans*. However, because these IS*2404* positive amoebae originated from both BU endemic and non-endemic areas, we remain sceptical about their implication in the transmission of *M. ulcerans* to humans.

## Introduction

Most mycobacteria are environmental opportunistic species that only occasionally infect humans [1]. Only few mycobacterial species are known to be obligate parasites, such as *Mycobacterium tuberculosis* and *M. leprae*, the causative agents of tuberculosis and leprosy respectively. The third most common mycobacterial disease, Buruli ulcer (BU), is caused by *M. ulcerans*, an environmental opportunistic mycobacterium. BU occurs mainly in rural areas of West and Central Africa with over 40,000 cases reported between 2002 and 2010 [2]. To the present day, the main reservoir of *M. ulcerans* and its transmission from the environment to humans remain unknown.

Epidemiological data from Africa suggest that proximity to slow-flowing or stagnant water best explains the distribution pattern of BU [3]. Nevertheless, it is unlikely that *M. ulcerans* occurs free-living in these waters, because (i) *M. ulcerans* evolved recently from the generalist, more rapid-growing environmental *M. marinum* via lateral gene transfer and reductive evolution to become adapted to a more protected niche [4], and (ii) since *M. ulcerans* is sensitive to several antibiotics, such as streptomycin and rifampicin, it is unlikely that the bacteria occur free living in an environment where *Streptomyces griseus* and *Amycolatopsis rifamycinica*, producers of respectively streptomycin and rifampicin, thrive [5], [6]. In order to survive without protection of a host a or biofilm, *M. ulcerans* would have developed natural resistance against these antibiotics as is the case for most opportunistic non tuberculous mycobacteria [6].

Recently in Australia, *M. ulcerans* has been found at high prevalence in two species of possum, thus suggesting a role for these mammals as reservoirs of *M. ulcerans* [7]. Although in Africa *M. ulcerans* has never been found in such high numbers in any particular element of the environment, low levels of *M. ulcerans* DNA were detected in many biotic components of aquatic ecosystems, such as plants, snail, fish and insects [3], indicating that *M. ulcerans* is ubiquitous in these ecosystems. The most explored hypothesis of *M. ulcerans* transmission in Africa argues that microphagous arthropods, e.g the heteropteran waterbugs, feed on *M. ulcerans* in water or biofilms, which are in turn consumed by predatory insects that may occasionally bite humans [8]. *M. ulcerans* bacilli are thought to be concentrated along this food chain, resulting in a sufficient infectious dose for humans [8]–[12]. Although the exact role of insects in the transmission of *M. ulcerans* remains to be proven [13], a series of experiments do support this hypothesis [9]–[12] and recent extensive fieldwork found a relatively high prevalence of *M. ulcerans* DNA in several waterbug species in a BU endemic area [14]. In addition, the only successful cultivation of *M. ulcerans* from an environmental source was from an aquatic hemipteran [15].

Nonetheless, these transmission hypotheses do not exclude an important role for other host species as *M. ulcerans* reservoirs. Recently, it has been postulated that amoebae might represent hosts for *M. ulcerans* and that they could be involved in the transmission from the environment to humans [3], [16], [17]. A study in Benin found that the detection frequency of free-living amoebae in water bodies in BU endemic villages was higher than in non-BU endemic villages [16]. *M. ulcerans* has been shown to form a biofilm on aquatic plants [18], and amoebae are often the main predators in biofilm communities. As a response to amoebal predation, many bacteria have acquired resistance to digestion in amoebal food vacuoles [19]. Also many mycobacterium species have been shown to survive and even thrive intracellularly in protozoa [19], [20]. Because of their hydrophobic cell wall, mycobacteria tend to attach to surfaces and are easily phagocytised by protozoa [21] and macrophages [22] and can even actively promote their entry into phagocytes [23]. Mycobacteria can use nutrients of protozoa as a food source and the intracellular life offers protection against harmful and fluctuating environmental influences, as protozoan cysts are remarkably resistant to extremes of temperature, drought and all kinds of biocides [24], [25]. However, few studies have investigated whether mycobacteria infect amoebae in their natural environment. Natural resistance to amoebae can have important consequences, as bacteria that infect and evade digestion in amoebae might use the same tools to enter and resist destruction within macrophages [19], [26], which have similar properties, as has been shown for *Legionella pneumophila* [27], an environmental opportunistic bacterium with amoebae as main reservoir. An intracellular stay in amoebae even enhances the virulence of *L. pneumophila* against mammalian cells [28]. This feature is not restricted to *Legionella*; *M. avium* passaged through *Acanthamoeba castellanii* is also more virulent towards a mouse model [29]. Even though *M. ulcerans* was long considered an extracellular pathogen, there is increasing evidence that the bacterium exhibits an important intracellular phase in neutrophils and macrophages during human infection (reviewed in [30]). So far only one study, published in 1978, has shown that *M. ulcerans* can be phagocytized and retained in an *Acanthamoeba*, yet this study provided only limited results [31].

In the present study we experimentally determined the capacity of *M. ulcerans* to infect *A. polyphaga*. We then further investigated the role of free-living amoebae as hosts for mycobacteria in their natural environment, including the potential of these protozoa as reservoirs for *M. ulcerans*.

## Materials and Methods

### Experimental infection of *A. polyphaga* with *M. ulcerans*

The *M. ulcerans* strains used were ITM 030216 (Benin), ITM 980912 (China), ITM 5114 (Mexico) and ITM 842 (Surinam) from the collection of the Institute of Tropical Medicine (ITM), Antwerp, Belgium.

A one week old, and therefore starving, axenic culture of *A. polyphaga* CCAP 1501/15 in PYG712 broth was adjusted to 10^5^ cells/mL and 1 mL transferred into each of the wells of a 24-well tissue culture plate. The amoebae monolayers were seeded with suspensions of *M. ulcerans* in triplicate at an approximate multiplicity of infection of 1 (*M. ulcerans* to *A. polyphaga*) and plates were incubated at 30°C. Three hours after infection, 20 µg/ml kanamycin was added to prevent extracellular growth of *M. ulcerans*. At times 3 h, 3 d, 7 d and 14 d after infection, the supernatant was aspirated and discarded. At each time point the medium of the unused wells was replaced by fresh PYG712 broth containing 20 µg/mL kanamycin.

To estimate the number of intracellular colony forming units (CFU) present inside amoebae at each time point the monolayer was suspended in 0.1% SDS in order to lyse the amoebae. The lysed amoebae suspension was transferred into a tube containing glass beads and vortexed. This suspension, as well as two 10-fold dilutions were inoculated on Löwenstein-Jensen (LJ) medium. The tubes were then incubated at 30°C and read after 6 weeks.

To localize intracellular bacteria, the amoeba monolayer was suspended in PBS and then processed for electron microscopy and for acid-fast staining using the Ziehl-Neelsen (ZN) method.

For transmission electron microscopy the processing of samples was carried out taking into account that bacteria, including mycobacteria, have specific requirements for adequate preservation as discussed elsewhere [32]. Briefly, a volume of 0.3 mL of the suspended monolayer was pelleted and the pellet was pre-fixed with 4% formaldehyde-1.25% glutaraldehyde-10 mM CaCl_2_ for 24 h, then fixed in 1% OsO4–10 mM CaCl_2_ for 16 to 24 h, and then post-fixed in aqueous 1% uranyl acetate for 1 h. Further processing for electron microscopy was carried out with ethanol dehydration and Epon embedding. Ultrathin sections were double-stained with uranyl acetate (saturated aqueous solution) for 5 min followed by lead citrate for 3 min.

### Detection and cultivation of mycobacteria and amoebae from the environment

#### Sample collection

**Figure 1:**
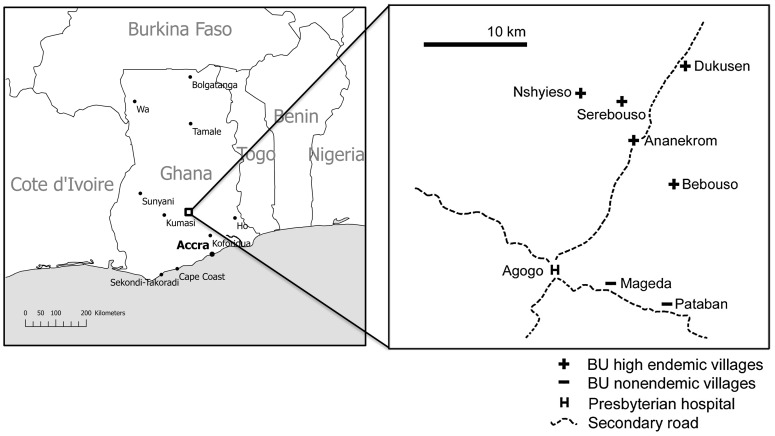
Map of the study area [**63**].

Water (n = 13), biofilm (n = 90) and aquatic detritus samples (n = 45) were collected in and around water bodies of seven communities around Agogo (Asante Akim North District, Ashanti Region, Ghana) (Figure 1). Five of these (Ananekrom, Nshyieso, Bebuso, Dukusen en Serebuoso) are BU endemic, with a village-based prevalence ranging from 0.47 to 2.14% in 2006 and 2007, while two communities (Mageda and Pataban) are considered non-endemic as no cases of BU were reported in these villages in the two years preceding this study. All water bodies sampled contained shallow, slow-flowing water that was largely shadowed by surrounding vegetation. People resident in these villages frequently come into contact with the water during walks, fishing and recreational activities and sometimes by using the water for cooking and drinking.

At every site, 3 L of water was sieved (pore size 100 µm) and filtered (pore size 0.45 µm) by use of a vacuum pump. Biofilm samples were taken with scalpels and cotton swabs from herbaceous plants (over a length of 20 cm along the stem) and wood pieces (10 cm^2^). Detritus was stored in 50 mL Falcon tubes.

#### Sample processing

Water filters were cut into small pieces in a Falcon tube, 15 mL PBS was added and the filtrand was suspended by shaking. Biofilms were suspended in 15 mL PBS in Falcon tubes by shaking. Detritus was transferred to stomacher bags, 50 mL 0.2% Tween80 in PBS was added and the bags were kneaded for 1 minute. The mixture was allowed to settle for 30 minutes after which 15 mL of the top layer was transferred to a 50 mL Falcon tube. These suspensions were used for cultivation of amoebae and mycobacteria (see below). DNA was extracted from these sample suspensions using a modified method of Boom [33], as described in [34].

#### Cultivation of intracellular and extracellular mycobacteria

From every suspended sample two 5 mL subsamples were prepared: one subsample was used for the isolation of extracellular mycobacteria and the second for the isolation of mycobacteria present intracellularly in microorganisms. To obtain extracellular mycobacterial cultures, the samples remained unprocessed until the decontamination procedure. To extract intracellular mycobacteria, we incubated the sample overnight in 40 µg/mL kanamycin, a mycobacteria-killing antibiotic that does not penetrate the cell membrane of eukaryotic organisms [16], [35]. The suspension was centrifuged for 5 minutes at 500 g and the pellet resuspended in 5 mL PBS with 0.5% SDS to lyse non-mycobacterial microorganisms. Decontamination by the oxalic acid method was performed as previously described on both sample parts [36]. After decontamination, the pellet was resuspended in 1 mL PBS of which 0.1 mL were inoculated on LJ medium. The inoculated media were incubated at 30°C for a maximum of twelve months and were read weekly. Smears of bacterial cultures were stained with the ZN method for the detection of acid-fast bacilli (AFB). The decontamination process was repeated when the culture consisted of a mixture of mycobacteria and other organisms. When the smear was composed of more than 80% mycobacteria, a loopful of the culture was suspended in 400 µL 1× TE buffer and heat-inactivated at 100°C for 5 minutes to release bacterial DNA. For the identification of the mycobacteria, these DNA extracts were subjected to a nested PCR targeting the 16S rRNA gene common to all mycobacteria [37]. The PCR products were sequenced using the second primer set by the VIB Genetic Service Facility (Antwerp, Belgium) and identified to the species level by performing a blast-search in NCBI GenBank.

#### Cultivation of amoebae

Non-nutrient agar plates coated with *Escherichia coli* served as medium for amoebal growth, as described by Page [38]. Briefly, about 1 cm^2^ piece of filter containing the water filtrand, about 0.5 cm^3^ of the detritus sample or a drop of the dissolved biofilm sample was placed on the centre of the agar plates. Plates were incubated at 30°C and read daily under a light microscope until amoebal growth was visible.

Morphologically different amoebae from the same sample were separated on different plates. To purify the amoeba cultures, contaminant-free portions of the agar plate were cut out and placed on a fresh plate. Such agar portions were also transported in 2 mL eppendorf tubes from Ghana to Belgium. When cultures were free of contaminants or pure amoeba cultures were impossible to obtain, the amoebae were scraped off the plate using 1.5 mL PBS. A few drops were fixed on a slide and stained by the ZN method to visualize AFB.

DNA from 250 µL of the amoeba suspension was extracted using the modified method of Boom [33], [34]. To detect mycobacterial DNA present in the amoeba cultures, the PCR assay targeting the 16S rRNA gene was performed as described above. To detect *M. ulcerans* DNA, a real-time PCR assay was performed as described below.

In order to identify amoeba species, three PCR assays were performed targeting members of the Vahlkampfiidae (using primer pairs JITSfw and JITSrv [39]), *Naegleria sp.* (using primer pairs ITSfw and ITSrv [40]) and *Acanthamoeba sp.* (using primer pairs JDP1 and JDP2 [41]). The PCR products were sequenced by the VIB (Flanders Institute for Biotechnology) Genetic Service Facility (Antwerp, Belgium) using the respective PCR primers. For *Acanthamoeba sp.*, the internal sequencing primer 892c was also used [41]. Sequences of Vahlkampfiidae (including *Naegleria sp.*) were blasted at the NCBI GenBank to search for similar known sequences. Sequences from *Acanthamoeba* were aligned with the DF3 fragment of the 15 known *Acanthamoeba* genotypes using Prankster [42].

#### Identification of *M. ulcerans*

For the detection of *M. ulcerans* in the samples and amoebae cultures, we performed two duplex real-time PCR assays on DNA extracted directly from the samples and on DNA extracted from the amoeba cultures isolated from these same samples. The PCR assays targeted the insertion sequence IS*2404* (a marker abundant in *M. ulcerans* but also present in some other mycobacteria [43]) and an Exogenous Internal Positive Control (Applied Biosystems) in the first assay, and IS*2606* and KR-B (the ketoreductase-B domain of the mycolactone polyketide synthase) in the second assay [44]. The reactions were executed in a 7300 Real-Time PCR System (Applied Biosystems) (at NMIMR, Legon, Ghana), Rotogene-3000 Real-Time Thermo cycler (Corbett Research) and iCycler (Bio-Rad) (at ITM, Antwerp, Belgium). Negative and positive controls for DNA extraction and PCR were included in every run and always had valid results.

#### Statistical analysis

With a test of equal proportions in the program R [45] we tested whether extracellular mycobacteria were isolated more frequently than intracellular mycobacteria.

Generalised mixed models for binomial distributions were constructed in R [45] to relate the relative occurrence of intracellular mycobacteria (number of intracellular cultures divided by number of both intracellular and/or extracellular cultures) or the isolation frequency of amoebae to BU endemicity and to the type of habitat (water, biofilm on herbs and on wood, and detritus). When necessary, we corrected for the possible effects of intrinsic differences between the different water bodies that were sampled, where the effect “water body” was nested in “BU endemicity” and was considered a random effect. The significance of the fixed effect of BU endemicity and the type of habitat was tested by constructing new linear models where these parameters were excluded, after which the new and the complete model were compared to each other using an Analysis of Variance (ANOVA). P-values lower than 0.05 were considered significant.

The significance of the effect of sampling different water bodies (a random effect nested in “BU endemicity”) was estimated using Bayesian statistics in the program WINBUGS [46], using 50000 iterations for the Markov Chain simulations. The generalised linear model and Bernouilli response distribution were constructed with the same parameters as described above and non-informative priors were used. Results were considered significant when the 95% confidence interval of the posterior distribution did not include zero.

## Results

### Co-incubation of *A. polyphaga* and *M. ulcerans* results in intracellular bacilli

**Figure 2:**
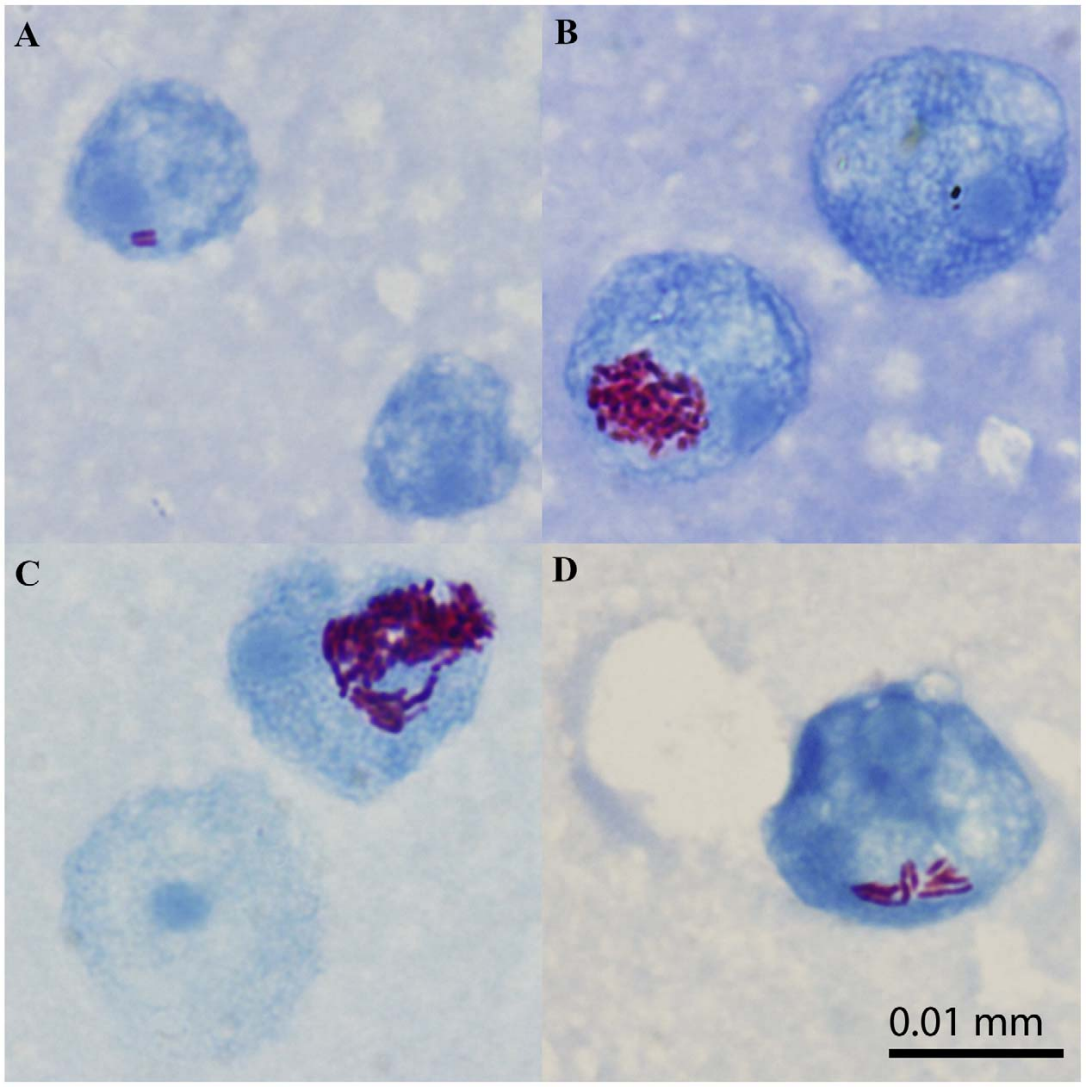
Light microscopic picture of *A. polyphaga* 3 hours after infection with *M. ulcerans* ITM 030216 (A), ITM 980912 (B), ITM 5114 (C), and ITM 842 (D).

**Figure 3:**
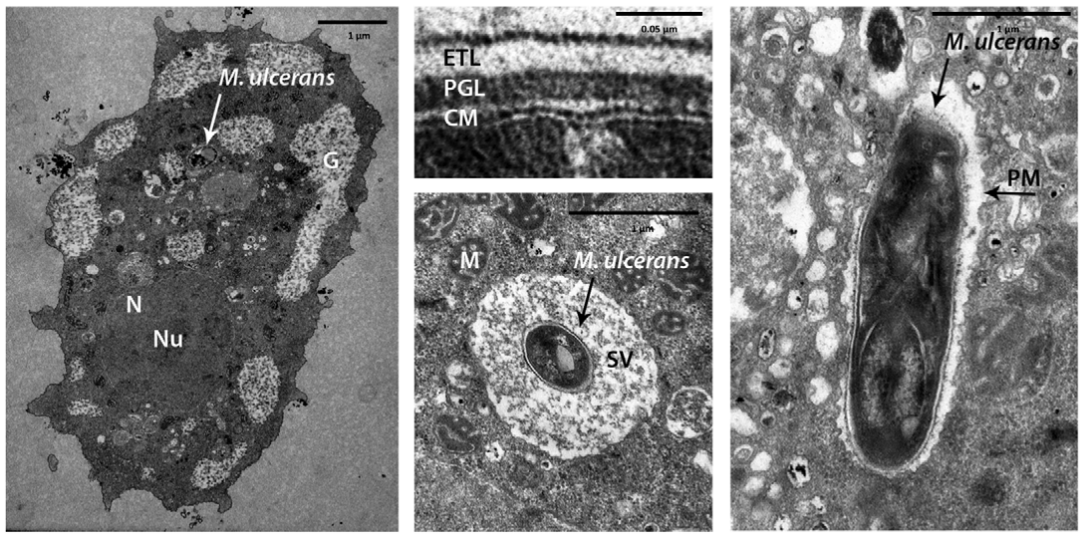
Transmission electron micrographs of *A. polyphaga* 3 hours after infection with *M. ulcerans* ITM 842. A: Intracellular localization of *M. ulcerans* within *A. polyphaga*; B: The cell envelope of *M. ulcerans*; C: *M. ulcerans* in a spacious vacuole; D: *M. ulcerans* in a tight vacuole. Nucleus (N), nucleolus (Nu), phagosomal membrane (PM), spacious vacuole (SV), and mitochondria (M) of *A. polyphaga*. Cell membrane (CM), electron transparent layer (ETL), and peptidoglycan layer (PGL) of *M. ulcerans*.

**Figure 4:**
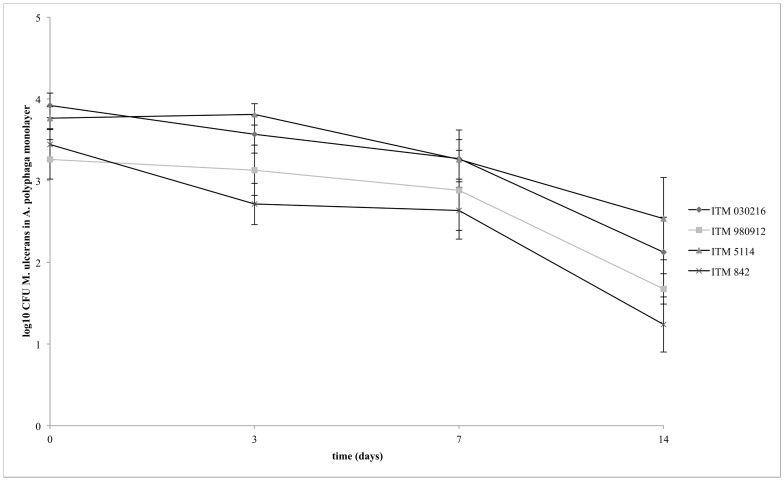
Viability of *M. ulcerans* (ITM 030216, ITM 980912, ITM 5114, and ITM 842) during infection of *A. polyphaga* at MOI 1.

Using light microscopy, AFB were observed co-localizing with amoebae after co-incubation of each *M. ulcerans* strain with *A. polyphaga* for 3 hours (Figure 2). Electron microscopy was used to confirm the intracellular localization (Figure 3). The bacilli were seen in phagocytic vacuoles with the phagosomal membrane tightly opposed to the bacillary surface (tight phagosomes) (Figure 3D) or, less frequently, inside “spacious vacuoles” (Figure 3C). The phagosomes contained single (Figure 3C and D) or groups of bacilli (not shown). About 45% of the bacilli in the electron micrographs looked normal according to the parameters previously defined [47], including the presence of an asymmetric profile of the cytoplasmic membrane with the outer layer thicker and denser than the inner layer (Figure 3B). As is typical of the ultrastructure of normal mycobacterial cell envelopes [48], an electron-transparent layer of the cell wall of *M. ulcerans* was observed (Figure 3B). No electron-transparent zone [49] was seen around the intracellular bacilli (Figure 3). Figure 4 shows that viable *M. ulcerans* persisted within *A. polyphaga* for the duration of the experiment (two weeks) although their numbers decreased with 1 to 2 log.

### Distribution of amoebae in natural water bodies in Ghana

181 amoeba cultures were obtained from 134 out of 148 collected samples (90.5%). The isolation frequency of amoebae did not differ significantly between BU endemic and non-endemic sites (p = 0.954, χ^2^_1_ = 0.004). Different habitats yielded different frequencies of amoeba isolation (p = 0.044, χ^2^_3_ = 8.084), with the highest detection frequency in detritus (97.8% vs. 76.9% in water, and 88.9% in biofilms). There was no significant difference between the sampled water bodies (estimated effect: 0.03; 95% CI: −0.19 to 0.24). Because 15 of the 181 amoeba cultures did not survive transportation and/or storage, mycobacteria were only searched for in the remaining 166 amoeba cultures (isolated from 124 different samples).

### Detection of *M. ulcerans* in samples and amoeba cultures

IS*2404* was detected by real-time PCR in 3 out of 148 samples, after extracting the DNA directly from these samples. In only one of them IS*2606* and KR-B were also detected, strongly suggesting the presence of *M. ulcerans* in that sample (Table 1). The Δ *C_T_* (IS*2606* - IS*2404*) value of 1.96 approaches the known fold difference in copy numbers between IS*2404* and IS*2606* for *M. ulcerans*, i.e. 2.3 [44]. However, the high *C_T_* values in all 3 of the positive assays (Table 1) imply the presence of less than a genome in the 1 µL DNA extract added to the PCR-mixture so that the Δ *C_T_*'s cannot be considered as true representations of the relative copy numbers of the repeated sequences. Therefore in the two IS*2404* positive yet IS*2606* and KR-B negative samples the presence of *M. ulcerans* cannot be denied nor confirmed.

**Table 1:** C_T_ values of samples and amoeba cultures positive for IS*2404* real-time PCR.

BU endemic (n = 116)					Non BU endemic (n = 32)				
DNA extracted from	Habitat	C_T_ IS*2404*	C_T_ IS*2606*	C_T_ KR-B	DNA extracted from	Habitat	C_T_ IS*2404*	C_T_ IS*2606*	C_T_ KR-B
Sample	Water	36.31	38.27	37.6	Sample	Biofilm on herbs	37.95	ND	ND
Sample	Biofilm on wood	38.45	ND	ND	Amoeba culture (92% identical to *Vahlkampfia inornata* * and *Acanthamoeba sp.* T4TD3)	Detritus	35.94	ND	ND
Amoeba culture (99% identical to *Vahlkampfia avara*)*	Biofilm on herbs	37.37	ND	ND	Amoeba culture (*Acanthamoeba sp.* T11TD3)	Detritus	37.48	ND	ND
Amoeba culture (*Acanthamoeba sp.* T5TD3)	Detritus	30.96	ND	ND					
Amoeba culture (*Acanthamoeba sp.* T11TD3)	Biofilm on wood	37.14	ND	ND					
Amoeba culture (99% identical to *Valkhampfiida avara* * and *Acanthamoeba sp.* T4TD3)	Detritus	35.73	ND	ND					
Dead amoeba culture	Biofilm on herbs	36.78	ND	ND					

ND = Not detected.

* Based on 340 bp of the internal transcribed spacer, including the 5.8S rRNA gene.

Out of the 166 amoeba cultures tested (originating from 124 different samples), seven were positive for IS*2404* (Table 1). Again, given the high *C_T_* values (Table 1), also here less than a genome was present in the 1 µL of DNA extract added to the PCR mixture. The IS*2404* positive amoeba cultures were isolated from BU endemic as well as BU non-endemic communities and from different microbial habitats. None of the IS*2404*-containing amoeba cultures tested positive for IS*2606* or KR-B. However, because of the low mycobacterial DNA content neither the absence or presence of *M. ulcerans* can be confirmed. None of IS*2404* positive amoeba cultures were isolated from samples that had already been found positive for IS*2404* in DNA extracted directly from the samples.

The following amoebae were identified among the IS*2404* positive cultures: *Vahlkampfia avara* (99% identical with the *V. avara* sequence in Genbank), a close relative of *V. inornata* (92% identical with the *V. inornata* sequence in Genbank), *A. lenticulata* (T5 genotype), *Acanthamoeba* sp. T11 genotype and *Acanthamoeba* spp. T4 genotype. One of the IS*2404* positive agar plates that supposedly supported an amoeba culture did not contain amoebae at the time of IS*2404* detection.

Identification of the IS*2404* negative amoebae will be detailed in a subsequent study by Amissah et al. (in preparation).

The geographical origins of the IS*2404* positive samples and amoeba cultures did not show any distribution pattern. We detected IS*2404* in at least 1 sample and/or amoeba culture from all sampled localities, except Bebuso.

### Mycobacteria are commonly found intracellularly in the environment and are often detected in amoeba cultures

As described in the methods section, subsamples were made to cultivate extracellular and intracellular mycobacteria. Twenty-six of the 148 samples were excluded from further analysis due to contamination of one or both of the subsamples. From 15 samples (12.2%) only intracellular mycobacteria were isolated, from 17 samples (13.9%) only extracellular mycobacteria, and from 32 samples (26.2%) both intra- and extracellular mycobacteria were isolated. Details are given in Table 2. In general the difference between the isolation frequency of extracellular and intracellular mycobacteria was not significant (χ^2^_1 = _0.17, p = 0.89). To assess whether the intracellular life style was more frequent in certain sites or certain habitats, we determined the relative isolation frequency of intracellular mycobacteria (i.e. the number of samples from which intracellular mycobacteria were cultivated divided by the total number of samples from which we cultivated mycobacteria –intracellular and/or extracellular), and related this to BU endemicity, sampling sites and habitat type. The relative isolation frequency of intracellular mycobacteria did not differ between BU endemic and non-BU endemic areas (0.77 vs. 0.68; p = 0.86, χ^2^_1_ = 0.03). The type of habitat, however, did have a significant effect on the relative occurrence of intracellular mycobacteria (p = 0.002; χ^2^_3_ = 15.1): intracellular mycobacteria were more frequently isolated from detritus samples (relative isolation frequency of 0.95) than from biofilm samples (relative isolation frequency of 0.63; p = 0.01).

**Table 2:** Overview of intracellular and extracellular mycobacteria isolated from different habitats in BU endemic and non-endemic areas.

	BU endemic area			Non BU endemic area		
	Intracellular/Sample size	Extracellular/Sample size	Intracellular/All cultures	Intracellular/Sample size	Extracellular/Sample size	Intracellular/All cultures
Water	1/10 (0.10)	2/10 (0.20)	1/2 (0.5)	0/2 (0.0)	0/2 (0.0)	0/0
Biofilm	17/65 (0.26)	22/65 (0.34)	17/27 (0.63)	8/18 (0.44)	9/18 (0.50)	8/13 (0.62)
Detritus	16/19 (0.84)	11/19 (0.58)	16/16 (1)	5/8 (0.63)	5/8 (0.63)	5/6 (0.83)
Total	34/94 (0.36)	35/94 (0.37)	34/44 (0.77)	13/28 (0.46)	14/28 (0.50)	13/19 (0.68)

Samples from which either the intracellular and/or extracellular cultures were contaminated, were discarded from this table and analyses. In the first and fourth column the number of samples from which we cultivated intracellular mycobacteria is given divided by the sample size. The same is presented in the second and fifth column, but with extracellular mycobacteria. In the third and sixth column, the relative isolation frequency of intracellular mycobacteria is given, calculated as the number of samples from which we cultivated intracellular mycobacteria divided by the total number of samples with mycobacteria cultures.

Based on a 821 to 837 bp portion of their 16S-rRNA gene sequence, 76 isolated mycobacteria (of intra- and extracellular origin) could be identified to the species level, with their sequence >99% identical to reference strains of which the sequence is present in GenBank. For 27 isolates, 16S rRNA-DNA sequence based identification was not possible due to the presence of a mixture of different species in the culture. An overview of the identified mycobacterial isolates is shown in Table 3 and Table S1. Species diversity did not show a marked difference between any type of isolation source (Table 3, S1).

**Table 3:** Identified mycobacteria species isolated from intracellular and extracellular sources.

Species (sequence similarity with reference strain in GenBank*)	Isolated from intracellular source	Isolated from extracellular source
*M. arupense* (99.5–100%)	22	27
*M. fortuitum* (99.5–100%)	5	6
*M. gordonae* (99.0%)	1	2
*M. peregrinum*/*septicum* (99.9–100%)	4	7
*M. chelonae/massiliense/abscessus* (99.9%)	1	0
*M. scrofulaceum* (99.5%)	0	1
Mixed, unidentified cultures	18	9
Contaminated cultures	12	22
Negative cultures	85	74

* Based on 821 to 837 bp of the 16S rRNA-gene.

Mycobacterial 16S-rRNA-DNA was detected in 29 amoeba cultures (17.5%), isolated from 25 out of 124 samples (20.2%). Mycobacterial presence was confirmed by microscopy in 13 of these positive cultures; 1 to 100 AFB were detected per 100 fields, which approximates to orders of 10^3^ to 10^5^ bacilli per culture of amoebae. No AFB were observed co-localising with the amoebae, however.

## Discussion

Amoebae are good candidates to be a reservoir of the elusive *M. ulcerans*, but this relationship has not yet been thoroughly investigated. Here, we study the potential for amoebae to host *M. ulcerans* both experimentally as by sampling an aquatic environment. Our results show that *M. ulcerans* can indeed be phagocytosed in vitro by *A. polyphaga* and that viable bacilli persist for at least 2 weeks. We observed both tight and spacious phagosomal vacuoles containing *M. ulcerans* in infected *A. polyphaga* with transmission electron microscopy, as has been described for *M. avium*-infected *A. castellanii* [29], [47] and for macrophages infected with mycobacteria [50], including *M. ulcerans* [51]. The observed reduction in the number of viable bacilli is probably due to bacilli that are expelled by the amoebae after a phase of intracellular multiplication, as has been reported for in vitro mycobacteria-infected macrophages [52], [53]. The kanamycin in the medium therefore probably killed released bacteria and resulted in an underestimation of the capacity of *M. ulcerans* to grow inside the protozoan cells. Compared to a noninfected *A. polyphaga* monolayer, infection with the *M. ulcerans* strains did not result in a higher loss of cells (data not shown) indicating that the infection did not affect *A. polyphaga* viability.

By analysing samples from an aquatic environment in BU endemic and nearby non-endemic communities in southern Ghana, we found several mycobacterium species intracellularly in eukaryotic micro-organisms. Most of the mycobacterium species we identified are potentially pathogenic to humans [54]–[57]. We did not isolate *M. ulcerans*, even not by successively passaging IS*2404* positive specimens and amoeba cultures in mouse footpads, the method that has led to the only successful isolation of *M. ulcerans* from the environment [15] (data not shown). We isolated mycobacteria as frequently from an intracellular source as free-living, suggesting that it is quite common for several species of mycobacteria to infect micro-organisms in natural circumstances. The intracellular lifestyle was found significantly more frequent in detritus samples compared to water and biofilm samples. This could be due to the low oxygen levels in this organic debris. For several environmental bacteria (including *M. avium*) it has been shown that oxygen depletion (and other conditions that typically dominate in animal intestines) triggers the invasion of and enhances the survival within host cells [58], [59].

We detected the marker IS*2404* in 1 water and 2 biofilm samples collected in a BU endemic and a nearby non-endemic community in southern Ghana. In addition, we detected the same marker in 6 amoeba cultures obtained from other samples. This is the first report of the detection of the marker IS*2404*, suggestive of *M. ulcerans* presence, in amoeba cultures isolated from the environment. It is noticeable that we tripled our detection frequency of IS*2404* by searching in the amoeba cultures in addition to the original samples. We could not observe AFB in the smears of these amoeba cultures, but one must take into account that *M. ulcerans* and other IS*2404* containing mycobacteria grow very slowly and thus were probably present in very low quantities on the amoeba cultures. On LJ-medium, *M. ulcerans* colonies only appear after an average of 10 weeks in primary culture from clinical specimens [60]. From environmental sources, *M. ulcerans* was only isolated once despite numerous attempts [15].

Other mycobacteria were also quite frequently detected in amoeba cultures (in 17.5%), by a PCR assay targeting their 16S-rRNA gene. For these, mycobacterial presence could be confirmed by microscopy in almost half of the positive cultures. However, the AFB were not observed inside or attached to the amoebae. The fact that in our study mycobacteria could still be detected after multiple subcultures of the amoeba cultures, suggests that the mycobacteria were multiplying extracellularly on the agar plates. IS*2404* was in fact also detected on one agar plate on which the amoebae did not survive subculturing. Similarly, in a co-culture study of *M. avium* and *A. polyphaga*, *M. avium* was shown to persist and multiply both intracellularly and extracellularly as a saprophyte on the excrement of *A. polyphaga*, and mycobacterial growth was most extensive extracellularly [47].

The successful uptake and persistence of *M. ulcerans* inside *A. polyphaga* in vitro and the higher detection frequency of IS*2404* in amoeba cultures as opposed to the crude samples from the environment suggest that amoebae may act as a host for *M. ulcerans* in natural circumstances. However, our data do not reveal a significant role for protozoa in the distribution patterns of BU disease in humans, so we remain sceptical about their involvement in the direct transmission of *M. ulcerans* to humans. If a protozoan were to be principally responsible for the observed distribution pattern of BU in humans, one would expect either a particular species with a limited distribution to harbour *M. ulcerans*, or otherwise several species that only do so in areas where BU actually occurs in humans. In this study, however, we detected IS*2404* as frequently in amoeba cultures isolated from BU endemic as from non-BU endemic communities. Moreover, 5 different protozoan species from two divergent families were identified in the IS*2404* positive amoeba cultures, some of which are known to be cosmopolitan. On the other hand, we cannot completely rule out that some or all of the IS*2404* we detected originated from different mycobacterial species than *M. ulcerans*.

More environmental research is needed in Africa if we want to understand the distribution of BU, and to prevent its transmission from the environment to humans. Environmental research of *M. ulcerans* has been severely hampered by the difficulties of detecting the pathogen in the environment. Our results indicate that perhaps amoeba cultures can serve for improved detection of *M. ulcerans* in environmental samples. Co-cultivation with an existing amoeba culture is a technique to selectively isolate amoebae-resistant bacteria that are difficult to grow from the environment [61] and has already been proven successful in the identification of new pathogens and their distribution patterns [62].

## Supporting Information

Table S1 **16S-rRNA gene sequence information of identified mycobacterial isolates.**
(DOCX)
