# Supplementary material for: Amoebae as Potential Environmental Hosts for Mycobacterium ulcerans and Other Mycobacteria, but Doubtful Actors in Buruli Ulcer Epidemiology
Source: PLoS Negl Trop Dis. 2012 Aug 7;6(8):e1764. doi: 10.1371/journal.pntd.0001764 (PMC3413716; doi:10.1371/journal.pntd.0001764)
Supplement: Table S1 — 16S-rRNA gene sequence information of identified mycobacterial isolates. (DOCX) [file pntd.0001764.s001.docx]

| Isolate nr. | Isolation source | Isolation locality | Species (% sequence similarity* with reference strain) | Accession number in Genbank with 100% sequence similarity* **°** |
| --- | --- | --- | --- | --- |
| ITM090670 | Biofilm - Extracellular | Ananekrom | *M. arupense* (100% AR30097) | NR043588.1 |
| ITM091097 | Biofilm - Extracellular | Ananekrom | *M. arupense* (100% AR30097) | NR043588.1 |
| ITM090623 | Biofilm - Extracellular | Ananekrom | *M. arupense* (100% AR30097) | NR043588.1 |
| ITM090666 | Biofilm - Extracellular | Ananekrom | *M. arupense* (100% AR30097) | NR043588.1 |
| ITM090659 | Biofilm - Intracellular | Ananekrom | *M. arupense* (100% AR30097) | NR043588.1 |
| ITM090671 | Biofilm - Intracellular | Ananekrom | *M. arupense* (100% AR30097) | NR043588.1 |
| ITM091099 | Detritus - Intracellular | Ananekrom | *M. arupense* (100% AR30097) | NR043588.1 |
| ITM090663 | Detritus - Intracellular | Ananekrom | *M. arupense* (100% AR30097) | NR043588.1 |
| ITM090627 | Water - Extracellular | Ananekrom | *M. arupense* (100% AR30097) | NR043588.1 |
| ITM091076 | Water - Intracellular | Ananekrom | *M. arupense* (100% AR30097) | NR043588.1 |
| ITM090672 | Water - Intracellular | Ananekrom | *M. arupense* (99.5 % AR30097) | ***JX119205*** |
| ITM090628 | Biofilm - Intracellular | Ananekrom | *M. fortuitum* (100% DSM44220) | FR733720.1 |
| ITM091073 | Biofilm - Extracellular | Ananekrom | M. gordonae (99.0 % ATCC14470) | GU142930.1 |
| ITM091081 | Detritus - Intracellular | Ananekrom | *M. septicum/peregrinum* (100 % ATCC700731) | NR042916.1 |
| ITM091080 | Detritus - Extracellular | Ananekrom | *M. septicum/peregrinum* (99.9 % ATCC700731) | ***JX119210*** |
| ITM090660 | Detritus - Intracellular | Bebuso | *M. arupense* (100% AR30097) | NR043588.1 |
| ITM090652 | Biofilm - Extracellular | Bebuso | *M. fortuitum* (100 % ATCC49404) | NR042914.1 |
| ITM091094 | Detritus - Extracellular | Bebuso | *M. septicum/peregrinum* (100 % ATCC700731) | NR042916.1 |
| ITM090880 | Biofilm - Extracellular | Dukusen | *M. arupense* (100% AR30097) | NR043588.1 |
| ITM090632 | Biofilm - Extracellular | Dukusen | *M. arupense* (100% AR30097) | NR043588.1 |
| ITM090661 | Biofilm - Extracellular | Dukusen | *M. arupense* (100% AR30097) | NR043588.1 |
| ITM090876 | Detritus - Extracellular | Dukusen | *M. arupense* (100% AR30097) | NR043588.1 |
| ITM090870 | Detritus - Extracellular | Dukusen | *M. arupense* (100% AR30097) | NR043588.1 |
| ITM090655 | Detritus - Intracellular | Dukusen | *M. arupense* (100% AR30097) | NR043588.1 |
| ITM090633 | Detritus - Intracellular | Dukusen | *M. arupense* (100% AR30097) | NR043588.1 |
| ITM090869 | Detritus - Intracellular | Dukusen | *M. arupense* (100% AR30097) | NR043588.1 |
| ITM090269 | Biofilm - Extracellular | Dukusen | *M. arupense* (99.9 % AR30097) | GU084182.2 |
| ITM090630 | Biofilm - Extracellular | Dukusen | *M. gordonae* (99.0 % ATCC14470) | GU142930.1 |
| ITM090270 | Biofilm - Intracellular | Dukusen | *M. gordonae* (99.0 % ATCC14470) | GU142930.1 |
| ITM091072 | Biofilm - Extracellular | Dukusen | *M. scrofulaceum* (99.5 % ATCC19981) | ***JX119208*** |
| ITM090653 | Biofilm - Intracellular | Dukusen | *M. chelonae/massiliense/abscessus* (99.88 % ATCC19237) | ***JX119201*** |
| ITM090651 | Detritus - Intracellular | Mageda | *M. arupense* (100% AR30097) | NR043588.1 |
| ITM090662 | Detritus - Extracellular | Mageda | *M. arupense* (99.6 % AR30097) | ***JX119198*** |
| ITM090093 | Biofilm - Extracellular | Mageda | *M. fortuitum* (99.9 % CIP104534) | JN049505.1 |
| ITM090875 | Biofilm - Extracellular | Mageda | *M. septicum/peregrinum* (100 % ATCC700731) | NR042916.1 |
| ITM091075 | Detritus - Intracellular | Mageda | *M. septicum/peregrinum* (100 % ATCC700731) | NR042916.1 |
| ITM090657 | Detritus - Extracellular | Mageda | *M. septicum/peregrinum* (99.9 % ATCC700731) | ***JX119202*** |
| ITM090665 | Biofilm - Intracellular | Nshieyso | *M. arupense* (100% AR30097) | NR043588.1 |
| ITM090625 | Biofilm - Extracellular | Nshyieso | *M. arupense* (100% AR30097) | NR043588.1 |
| ITM091093 | Detritus - Extracellular | Nshyieso | *M. arupense* (100% AR30097) | NR043588.1 |
| ITM091098 | Detritus - Intracellular | Nshyieso | *M. arupense* (100% AR30097) | NR043588.1 |
| ITM091091 | Detritus - Intracellular | Nshyieso | *M. arupense* (99.6 % AR30097) | ***JX119198*** |
| ITM090656 | Biofilm - Extracellular | Nshyieso | *M. arupense* (99.9 % AR30097) | FJ538896.1 |
| ITM090654 | Biofilm - Intracellular | Nshyieso | *M. arupense* (99.9 % AR30097) | GU084182.2 |
| ITM091087 | Biofilm - Intracellular | Nshyieso | *M. septicum/peregrinum* (99.9 % ATCC700731) | ***JX119210*** |
| ITM090626 | Biofilm - Intracellular | Pataban | *M. arupense* (100% AR30097) | NR043588.1 |
| ITM091096 | Detritus - Extracellular | Pataban | *M. arupense* (100% AR30097) | NR043588.1 |
| ITM090649 | Biofilm - Extracellular | Pataban | *M. arupense* (99.5 % AR30097) | FJ538897.1 |
| ITM090877 | Biofilm - Extracellular | Pataban | *M. arupense* (99.5 % AR30097) | FJ538898.1 |
| ITM090874 | Detritus - Extracellular | Pataban | *M. arupense* (99.5 % AR30097) | FJ538897.1 |
| ITM090673 | Detritus - Intracellular | Pataban | *M. arupense* (99.8 % AR30097) | ***JX119200*** |
| ITM090674 | Biofilm - Extracellular | Pataban | *M. arupense* (99.9 % AR30097) | ***JX119206*** |
| ITM090881 | Detritus - Extracellular | Pataban | *M. arupense* (99.9 % AR30097) | ***JX119206*** |
| ITM090092 | Biofilm - Extracellular | Pataban | *M. fortuitum* (100% CIP104534) | NR042912 |
| ITM090884 | Biofilm - Intracellular | Pataban | *M. fortuitum* (100% DSM44220) | FR733720.1 |
| ITM090658 | Biofilm - Extracellular | Pataban | *M. fortuitum* (99.5 % CIP104534) | ***JX119203*** |
| ITM090629 | Biofilm - Intracellular | Pataban | *M. fortuitum* (99.8 % CIP104534) | ***JX119199*** |
| ITM090664 | Detritus - Extracellular | Pataban | *M. septicum/peregrinum* (100 % ATCC700731) | NR042916.1 |
| ITM090668 | Biofilm - Extracellular | Serebuoso | *M. arupense* (100% AR30097) | NR043588.1 |
| ITM090267 | Biofilm - Extracellular | Serebuoso | *M. arupense* (100% AR30097) | NR043588.1 |
| ITM090669 | Biofilm - Extracellular | Serebuoso | *M. arupense* (100% AR30097) | NR043588.1 |
| ITM090873 | Biofilm - Intracellular | Serebuoso | *M. arupense* (100% AR30097) | NR043588.1 |
| ITM090871 | Detritus - Extracellular | Serebuoso | *M. arupense* (100% AR30097) | NR043588.1 |
| ITM090631 | Detritus - Intracellular | Serebuoso | *M. arupense* (100% AR30097) | NR043588.1 |
| ITM090667 | Biofilm - Extracellular | Serebuoso | *M. arupense* (99.6 % AR30097) | ***JX119204*** |
| ITM090624 | Biofilm - Intracellular | Serebuoso | *M. arupense* (99.6 % AR30097) | ***JX119198*** |
| ITM091095 | Biofilm - Intracellular | Serebuoso | *M. arupense* (99.8 % AR30097) | ***JX119200*** |
| ITM090650 | Biofilm - Intracellular | Serebuoso | *M. arupense* (99.8 % AR30097) | ***JX119200*** |
| ITM090675 | Biofilm - Extracellular | Serebuoso | *M. arupense* (99.9 % AR30097) | ***JX119207*** |
| ITM090268 | Detritus - Extracellular | Serebuoso | *M. fortuitum* (100% DSM44220) | FR733720.1 |
| ITM090094 | Detritus - Extracellular | Serebuoso | *M. fortuitum* (100% DSM44220) | FR733720.1 |
| ITM091074 | Detritus - Intracellular | Serebuoso | *M. fortuitum* (100% DSM44220) | FR733720.1 |
| ITM090878 | Biofilm - Intracellular | Serebuoso | *M. fortuitum* (99.9 % CIP104534) | ***JX119197*** |
| ITM090879 | Biofilm - Extracellular | Serebuoso | *M. septicum/peregrinum* (100 % ATCC700731) | NR042916.1 |
| ITM091085 | Detritus - Intracellular | Serebuoso | *M. septicum/peregrinum* (100 % ATCC700731) | NR042916.1 |
| ITM091079 | Detritus - Extracellular | Serebuoso | *M. septicum/peregrinum* (99.9 % ATCC700731) | ***JX119209*** |

* based on a 821-837 bp portion of the 16S rRNA gene

° When the sequence of an isolate is 100% identical to the mentioned reference strain, the accession number of this strain is given. Otherwise, the accession number of a non-reference strain sequence that shares 100% 16S rRNA sequence similarity with our isolate is provided. If no sequence with 100% similarity to our isolates’ sequence was available in GenBank, we deposited this sequence in GenBank. The accession numbers of sequences derived from isolates from this study are in bold and italic.
